# Distinct Strategies Regulate Correlated Ion Channel mRNAs and Ionic Currents in Continually versus Episodically Active Neurons

**DOI:** 10.1523/ENEURO.0320-24.2024

**Published:** 2024-11-12

**Authors:** Jose A. Viteri, Simone Temporal, David J. Schulz

**Affiliations:** Division of Biological Sciences, University of Missouri-Columbia, Columbia, Missouri 65211

**Keywords:** central pattern generator, stomatogastric

## Abstract

Relationships among membrane currents allow central pattern generator (CPG) neurons to reliably drive motor programs. We hypothesize that continually active CPG neurons utilize activity-dependent feedback to correlate expression of ion channel genes to balance essential membrane currents. However, episodically activated neurons experience absences of activity-dependent feedback and, thus, presumably employ other strategies to coregulate the balance of ionic currents necessary to generate appropriate output after periods of quiescence. To investigate this, we compared continually active pyloric dilator (PD) neurons with episodically active lateral gastric (LG) CPG neurons of the stomatogastric ganglion (STG) in male *Cancer borealis* crabs. After experimentally activating LG for 8 h, we measured three potassium currents and abundances of their corresponding channel mRNAs. We found that ionic current relationships were correlated in LG's silent state, but ion channel mRNA relationships were correlated in the active state. In continuously active PD neurons, ion channel mRNAs and ionic currents are simultaneously correlated. Therefore, two distinct relationships exist between channel mRNA abundance and the ionic current encoded in these cells: in PD, a direct correlation exists between *Shal* channel mRNA levels and the A-type potassium current it carries. Conversely, such channel mRNA–current relationships are not detected and appear to be temporally uncoupled in LG neurons. Our results suggest that ongoing feedback maintains membrane current and channel mRNA relationships in continually active PD neurons, while in LG neurons, episodic activity serves to establish channel mRNA relationships necessary to produce the ionic current profile necessary for the next bout of activity.

## Significance Statement

Motor neurons must coregulate their ionic currents to ensure output stability. In neurons that are continually active, one possible strategy to achieve this involves using activity-dependent feedback to consistently maintain correlated levels of ion channel mRNAs underlying correlations among the corresponding ionic currents. However, neurons with transient periods of activity must use other strategies. We show that in episodically active neurons, ion channel mRNAs and the corresponding ionic currents are correlated in different states of activity. We propose that the temporal uncoupling between correlated mRNAs and currents in these cells allows episodically active neurons to stabilize the appropriate coregulated ionic currents even during periods of inactivity.

## Introduction

Central pattern generators (CPGs) drive rhythmic motor outputs that can be continually or episodically active (Golowasch, 2019). Episodically active CPGs drive behaviors like feeding (Sasaki et al., 2013), locomotion (Cazalets et al., 1996), and escape (Sakurai et al., 2014) that have periods of activity and inactivity. Conversely, CPGs also underlie continuous motor behaviors such as breathing (Santin et al., 2017) and some invertebrate cardiac rhythms (Saver et al., 1999). To maintain reliable neuronal outputs and behaviors, motor networks rely on preserving the stability of coregulated ionic currents (Hudson and Prinz, 2010; Zhao and Golowasch, 2012; Tran et al., 2019), with motor networks utilizing diverse strategies to do so (Temporal et al., 2012; Santin and Schulz, 2019; Viteri and Schulz, 2023). While continually active networks utilize activity-dependent feedback to maintain their underlying properties (Santin and Schulz, 2019), episodically active neurons in their inactive state must already possess the appropriate balance of ionic currents necessary to resume their normal outputs. Thus, mechanisms must exist that establish and maintain coregulated ionic currents in these neuron types in the absence of continuous activity-dependent feedback.

To investigate these mechanisms, we use the stomatogastric ganglion (STG) of the Jonah crab (*Cancer borealis*) which contains two motor networks working in tandem, each of which possesses a distinct firing pattern that models continual and episodic network activity. The pyloric network is a continually active set of neurons that control the dilation of the pylorus that filters ground food (Marder and Bucher, 2007), while the gastric mill network drives the movement of stomach teeth that grind ingested food (Stein, 2009), and is active (episodically) only when food is present. Changes in environmental temperature (Hernández-Sandoval et al., 2018), food availability (Scharf, 2016), or life cycle events like molting (Sugumar et al., 2013) can drastically reduce crustaceans feeding behavior for weeks to months (Scharf, 2016).

Previous work has shown that pyloric dilator (PD) neurons stabilize their outputs by sensing their ongoing activity (i.e., membrane voltage) to correlate their ion channel mRNAs (Santin and Schulz, 2019), which are simultaneously coupled with correlations of the corresponding ionic currents (Temporal et al., 2012). Conversely, we recently demonstrated that when the lateral gastric (LG) neuron of the gastric mill network becomes active, many new correlated ion channel mRNA relationships form that are not present in the inactive state of these cells (Viteri and Schulz, 2023). However, upon activation, those LG neurons must already possess appropriately balanced and correlated ionic currents allowing for normal patterned output. Taken together, we now hypothesize that while continually active neurons like PD couple the relationships between their channel mRNAs and ionic currents, a temporal uncoupling of this process may occur in episodically active cells like LG, whereby feedback during one cycle of activity establishes correlated mRNA relationships that are then used to constrain ionic currents for the next network activation after potentially long periods of inactivity.

**Figure 1.**
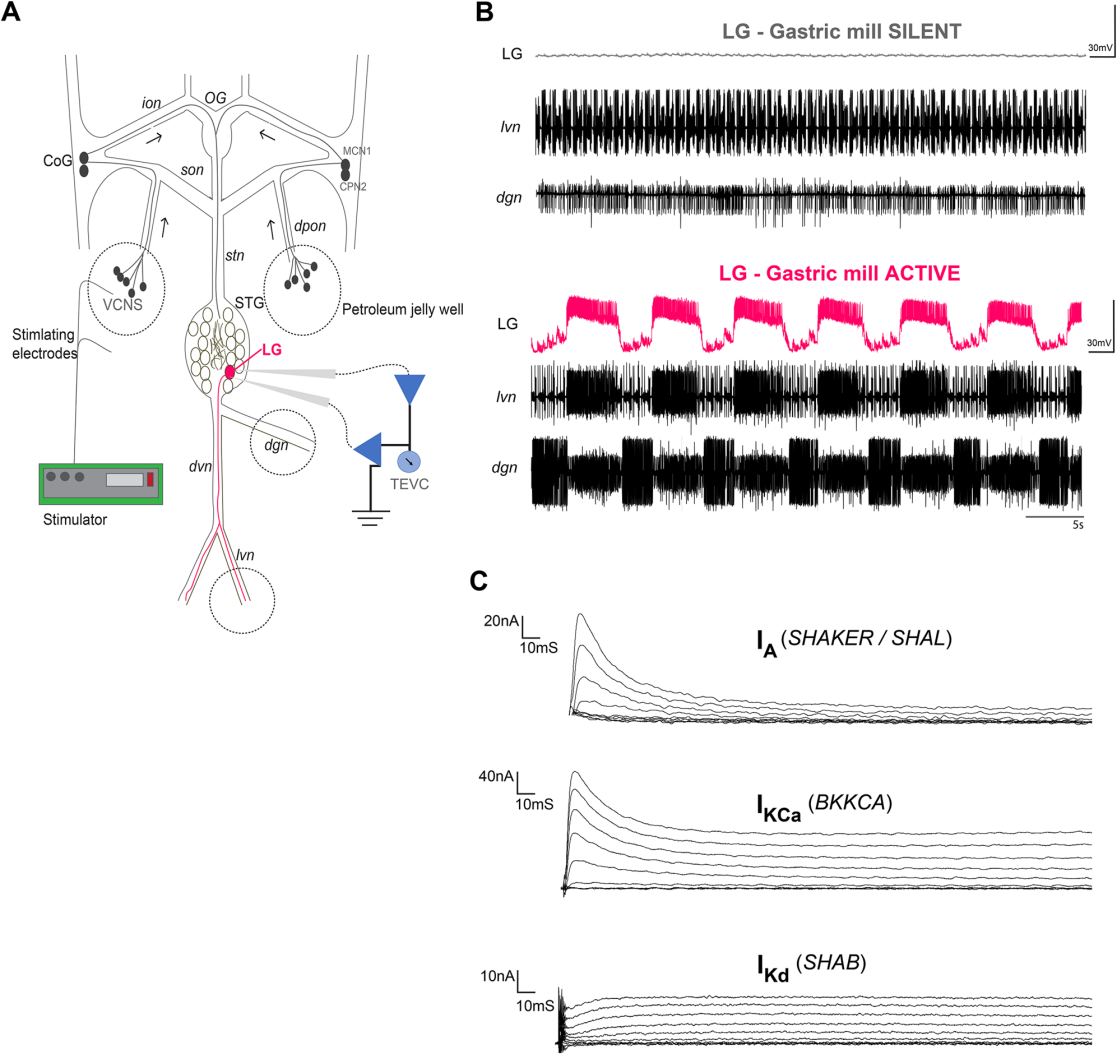
Activation of the lateral gastric (LG) neuron's active state via stimulation of descending inputs to the STG. ***A***, Schematic of the stomatogastric nervous system from the crab *Cancer borealis*. All descending modulatory inputs were preserved: the oesophageal ganglion (OG) and the commissural ganglia (CoG) provide descending modulatory inputs to the STG via the stomatogastric nerve (*stn*). The lateral gastric (LG) neuron used in these experiments is highlighted in the STG. A stimulator (A-M Systems) provided the necessary voltage (10–15 V) to experimentally turn on the gastric mill rhythm. This was accomplished by surrounding the dorsal posterior oesophageal nerves (*dpons*) with a petroleum jelly well and stimulating the *dpon* nerves. This stimulates the ventral cardiac neurons which in turn activate MCN1 and CPN2 neurons in the CoG. This induces the release of the *Cancer borealis* tachykinin-related peptide Ia (CabTRP Ia) which converges to LG and initiates LG's active state. ***B***, Representative recordings of LG's different states when the gastric mill is silent or active. Recordings of LG are taken from the same neuron before and after activation of the gastric mill rhythm. Recordings were taken extracellularly from the *lvn* and *dgn* nerves of the stomatogastric nervous system (STNS). Recordings from the *lvn* allow for visualization of the pyloric rhythm while recordings from the *dgn* allow for visualization of the gastric mill rhythm. LG axons run through the *lvn* and allow for confirmation of LG's active state during gastric mill activity. Calibration: 30 mV and 5 s. ***C***, Ionic currents measured in silent and active LG neurons. Ionic current magnitudes in LG neurons were measured at 0 mV on an IV plot generated from the current traces. In silent LG neurons, currents were measured acutely. In active LG neurons, currents were measured after 8 h of activity. The A-type potassium current (*I*_A_) was measured by subtracting the high threshold potassium current (*I*_HTK_) from an *I*_A_ TEVC protocol with a holding potential of −80 mV and 10 voltage steps from −60 to +30 mV (10 mV intervals). *I*_HTK_ was measured by using a leak-subtracted TEVC protocol with a holding potential of −40 mV and 10 voltage steps from −60 to +30 mV (10 mV intervals). The calcium-activated potassium current (*I*_KCA_) was measured by subtracting postcadmium (250 µM CdCl_2_) I_HTK_ current traces from precadmium *I*_HTK_ current traces. The delayed rectifier potassium current (*I*_Kd_) was measured by running the *I*_HTK_ TEVC protocol after the application of cadmium (250 µM CdCl_2_) to block *I*_KCa_.

Based on these hypotheses, we predicted a direct correlation between channel mRNA abundances and the resulting membrane currents in continually active pyloric neurons like PD. Conversely, in LG, we predicted no such correlation, as this relationship would be temporally uncoupled and no longer evident in such simultaneous measurements. To test this, we activated the gastric mill network (Beenhakker et al., 2004) and measured three potassium (K^+^) currents in LG neurons that had been active for 8 h and in control inactive LG neurons (Fig. 1). We then immediately collected those LG neurons and used quantitative RT-PCR (qPCR) to measure the abundances and pairwise relationships of the transcripts corresponding to the three K^+^ currents. We directly compared these results with data collected similarly from PD neurons.

## Materials and Methods

### Experimental model and subject details

We purchased adult male Jonah crabs (*Cancer borealis*) from the Fresh Lobster company. Crabs were maintained in artificial seawater chilled to 12°C. We anesthetized crabs in ice for 30 min and then removed the foregut and dissected out the stomatogastric nervous system (STNS). For each dissection, we kept intact the commissural ganglia (CoG), oesophageal ganglia (OG), inferior oesophageal nerves (ion), and stomatogastric ganglion (STG). All STGs were desheathed using a steel wire pin. STNS preparations were bathed in chilled physiological saline (12°C) with the following concentrations (in mM): 440 NaCl, 11 KCl, 13 CaCl_2_, 26 MgCl_2_, and 10 HEPES buffer, pH 7.45.

### Electrophysiological recordings and manipulations

We recorded pyloric and gastric mill activity by placing stainless steel electrodes in petroleum jelly wells built around the *lvn* and *dgn* nerves of our STNS preparations (Fig. 1*A*). Extracellular signals from pyloric and gastric mill activity were then amplified with an A-M Systems Model 1700 extracellular amplifier (A-M Systems). We identified LG neurons by comparing their intracellular activity with spikes on extracellular traces of the *lvn* and by using standard cell mapping procedures (Weimann et al., 1991; Beenhakker et al., 2004). Intracellular recordings from LG neurons were made using a 10–30 MΩ glass microelectrode filled with (in mM) 600 K_2_SO_4_ and 20 KCl and amplified using an Axoclamp 2B intracellular amplifier (Molecular Devices). We acquired all data with a Digidata 1322A digitizer (Molecular Devices).

Gastric mill network activity was initiated by placing stainless steel electrodes in petroleum jelly wells built around both dorsal posterior oesophageal nerves (*dpons*) and then passing voltage at 10−15 V rhythmic stimulus trains (10 × 6 s burst delivered at 0.06 Hz; 15 Hz intraburst stimulation rate; Beenhakker et al., 2004), with each stimulation event lasting ∼1 min and 30 s. The stimulation protocol was carried out using an A-M Systems isolated pulse stimulator Model 1200 (A-M Systems). Upon stimulation, preparations displayed robust gastric mill activity (Fig. 1*B*), including LG neuron bursting. Gastric mill activity lasted between 30 and 45 min. We reinduced gastric mill activity only when activity ceased as needed, such that we sustained LG's gastric mill activity for a period of 8 h.

Measurements of membrane currents were performed using two-electrode voltage clamp and always in the presence of 10^−6^ M tetrodotoxin (TTX; Sigma-Aldrich) to block voltage-gated Na^+^ channels. Current injection glass electrodes filled with (in M) 3 KCl had a resistance of 10−20 MΩ and voltage recording glass electrodes filled with (in mM) 600 K_2_SO_4_ and 20 KCl had a resistance of 30−40 MΩ. Input resistance for all LG neurons was 4−8 MΩ. Two-electrode voltage-clamp (TEVC) protocols were created, driven, and recorded with Clampex software (Molecular Devices). These voltage-clamp protocols were modified from those used previously in crustacean motor neuron preparations (Golowasch and Marder, 1992; Khorkova and Golowasch, 2007; Ransdell et al., 2012; Temporal et al., 2012). The mixed high threshold K^+^ current (*I*_HTK_) was measured by using a leak-subtracted TEVC protocol with a holding potential of −40 mV and 10 voltage steps from −60 to +30 mV (10 mV intervals). The transient A-type potassium current (*I*_A_) was measured by subtracting the high threshold potassium current (*I*_HTK_) from a TEVC protocol with a holding potential of −80 mV and the same 10 voltage steps from −60 to +30 mV (10 mV intervals). The delayed rectifier potassium current (*I*_Kd_) was measured by running the leak-subtracted *I*_HTK_ TEVC protocol after the application of cadmium (30–45 min of 250 µM CdCl_2_). The calcium-activated potassium current (*I*_KCA_) was measured by subtracting postcadmium (250 µM CdCl_2_) *I*_HTK_ current traces from precadmium *I*_HTK_ current traces. All current magnitude data shown were measured at the raw peak amount of current elicited at a holding potential of 0 mV.

### Experimental groups

We collected LG and PD neurons from two experimental groups that represent different states of ongoing activity. (1) Control preparations were isolated STNS that had intact ganglia and neuromodulatory inputs (*ion* nerves were intact) and were maintained in chilled physiological saline. Spontaneous pyloric activity was present, but we did not initiate any gastric mill activity (Fig. 1*B*). No spontaneous gastric mill activity was detected, and animals had not been fed for at least 72 h prior to the experiment. Hence, we considered these LG neurons to be “silent,” while the PD neurons from these preparations were considered “active.” Neurons from these controls were collected either immediately after identification or after completion of voltage-clamp measurements. (2) To collect LG neurons that had experienced ongoing activity, STNS preparations had intact ganglia and neuromodulatory inputs and experienced 8 h of ongoing stimulated gastric mill activity as described above (Fig. 1*B*). We considered these LG neurons to be “active.” We did not collect PD neurons from these stimulated preparations, as there are no significant differences in PD activity, or mRNA levels or relationships in PD cells from networks with ongoing gastric mill activity (Viteri and Schulz, 2023). Because voltage clamping requires the silencing of activity in both the control preparations and after gastric mill activation, all neurons that were harvested subsequent to voltage-clamp measurements experienced a period of inactivity prior to their final collection. This period was not longer than 90−120 min.

### Harvesting of identified neurons

Cell harvesting was performed as described previously (Schulz et al., 2007, 2006). At the conclusion of the experiment, a petroleum jelly well was built around the STG containing physiological saline. Subsequently, 2.5 mg/ml of protease (P6911, Sigma-Aldrich) was then directly added to the petroleum jelly well in order to digest and loosen connective tissue around the neurons of the STG. We then replaced the saline and protease in the well with fresh physiological saline which stopped digestion. We then incrementally substituted the fresh saline with cold ethylene glycol (EG; 70% EG and 30% physiological crab saline) over a period of 15 min. STG preparations were then stored in a −20°C freezer to further inhibit any cellular processes and prepare the cells for collection. After 1 h we pulled either LG or PD neurons using fine handheld forceps and placed each neuron into 400 µl of RNA lysis buffer (Zymo Research) and stored at −80°C.

### cDNA synthesis and preamplification of cDNA targets

RNA isolation and cDNA synthesis were performed using standard methods described in previous work (Northcutt et al., 2019). We used the Quick-RNA MicroPrep kit (Zymo Research) per the manufacturer's instructions to purify and isolate total LG RNA. We then reverse transcribed RNA using a mixture of oligo-dT and random hexamer primers (qScript cDNA SuperMix; Quantabio). Eight microliters of cDNA was then preamplified using PerfeCTa PreAmp SuperMix (Quantabio) according to the manufacturer's instructions (24 µl reaction volume). We used a 14-cycle PCR preamplification reaction protocol which was primed with a pool of target-specific primers (Northcutt et al., 2019). Seventy-six microliters of nuclease-free water was then used to dilute each preamplified sample to 100 µl total volume.

### Quantitative reverse transcription polymerase chain reaction (qRT-PCR)

We designed and validated TaqMan probes and primer sets (Extended Data Table 1-1) for the following voltage-gated ion channel genes: the delayed rectifier K^+^ channel *SHAB*, the A-type K^+^ channel *SHAKER*, the A-type K^+^ channel *SHAL*, and the large conductance Ca^2+^-activated K^+^ channel *BKKCA*.

10.1523/ENEURO.0320-24.2024.t1-1 Table 1-1 Primers and probes used for multiplex RT-QPCR reactions used in LG neurons. Download Table 1-1, DOCX file.

To further validate the correct identification of LG and PD neurons, we assayed harvested neurons for the presence of vesicular acetylcholine transporter (vAChT), choline acetyltransferase (ChAT), and vesicular glutamate transporter (vGluT). LG neurons are glutamatergic and thus express vGluT and lack expression of ChAT and vAChT, while the inverse is true of cholinergic PD neurons (Marder and Bucher, 2007; DeLong et al., 2009). Any putative LG neuron that lacked vGluT and/or highly expressed ChAT and vAChT was discarded from any further analysis. Similarly, any putative PD neuron that lacked ChAT and vAChT and/or highly expressed vGluT was discarded from further analysis.

Primer and probe sequences, as well as working concentrations, are as reported in a previous study (Northcutt et al. 2019). To make a 32 µl reaction supermix, we included the following: (1) 5 µl of preamplified template; (2) 6.4 µl iQ Multiplex Powermix (Bio-Rad Laboratories); (3) 1.6 µl of primer mix (IDT Integrated DNA Technologies) which contained 50 µM of forward and reverse primer for each gene; and (4) 1 µl of 10 µM dual-labeled Black Hole Quencher probe (LGC Biosearch Technologies). We then divided this supermix into 3 × 10 µl triplicates. In the end, this produced single qPCR reactions made up of 10 µl which were then loaded into a single well on a 96-well plate. The final primer concentration of each multiplex qPCR reaction was 2.5 µM and 0.3125 µM for each probe. All reactions were run on a CFX96 Touch Real-Time PCR Detection System (Bio-Rad Laboratories) with these cycling conditions: 95°C for 3 min, 40 cycles of 95°C for 15 s, and 58°C for 1 min. Fluorescence measurements were taken at the end of each cycle.

We used standard curves that were developed for each qPCR multiplex assay to assess the absolute quantification of mRNA abundances. To achieve this, we serially diluted custom gBlock gene fragments (Integrated DNA Technologies), from 1 × 10^6^ to 1 × 10^1^ copies for each reaction assay to define the upper and lower bounds of transcript detection. We calculated absolute abundances for target transcripts by using the efficiency and slope of standard curves, as well as accounting for the 14-cycle preamplification and dilution of cDNA templates described above.

### Experimental design and statistical analysis

All statistical analyses and data visualizations were performed using GraphPad Prism 10 [GraphPad Version 10.1.1 (270) Software]. For all LG and PD ion channel mRNA abundances and ionic current magnitudes, we used a Shapiro–Wilk test for normality to determine whether residuals for each distribution were normally distributed (*p* > 0.05). We compared the LG mRNA abundances and ionic current magnitudes of silent neurons with active neurons using a Welch's two-sample independent *t* test for normally distributed data (Shapiro–Wilk *p* > 0.05) and a Wilcoxon rank sum test on non-normal distributions (Shapiro–Wilk *p* < 0.05). For LG and PD channel mRNA and ionic current magnitude pairwise relationships, we used Pearson’s or Spearman correlation tests, depending on whether that distribution was normally distributed or not respectively (Shapiro–Wilk test for normality). We applied a Grubbs test for outliers on all distributions. We arbitrarily classified the strength of an ion channel mRNA or membrane current correlation with terminology carried over from previously published work (Schulz et al., 2007; Viteri and Schulz, 2023). If the Pearson’s or Spearman value was greater than an absolute value of 0.6 with a *p*-value of <0.05, the correlation was considered “strong.”

**Figure 2.**
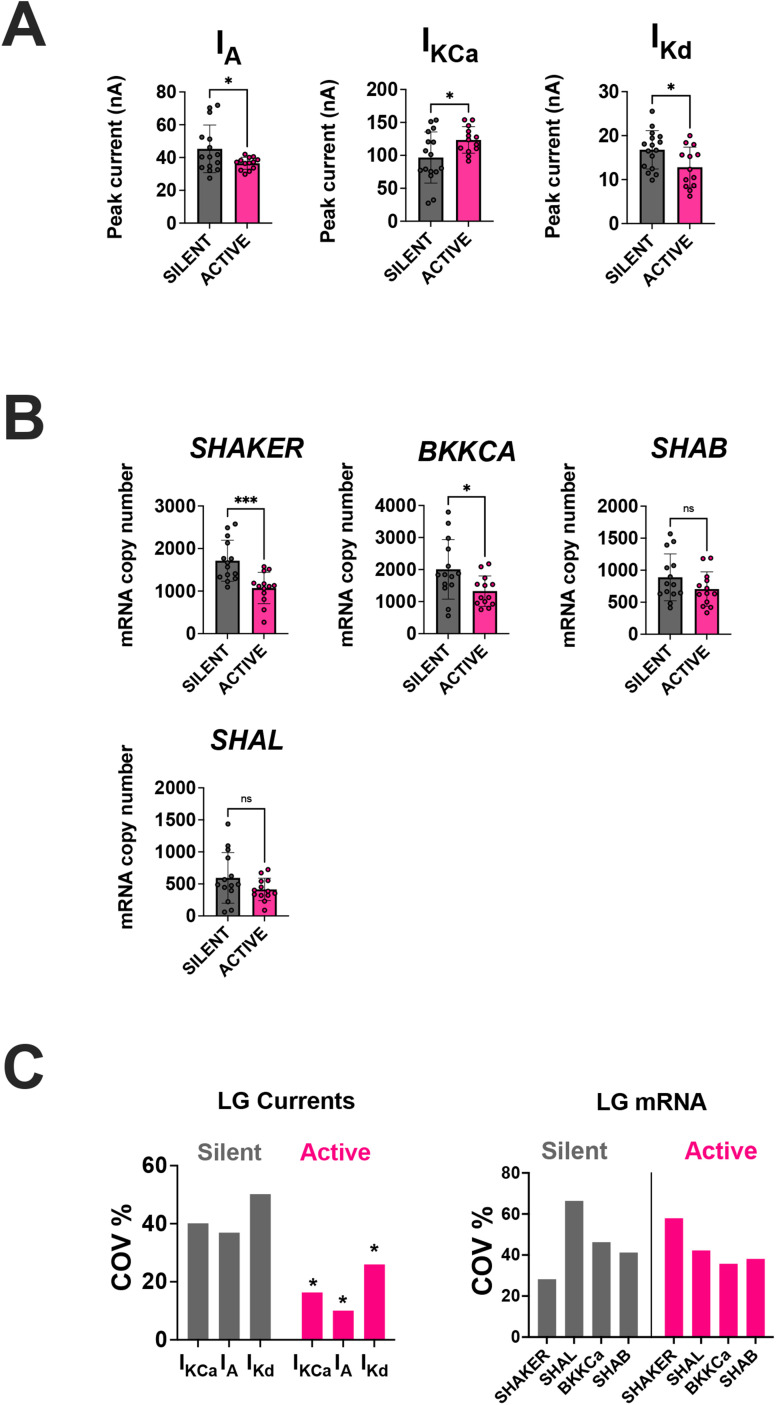
The active state of LG neurons induces changes in peak ionic current magnitudes and changes in corresponding ion channel mRNA abundances. ***A***, Bar graphs (mean ± SD) displaying the peak ionic current magnitudes for three of the potassium currents that were measured. Each point corresponds to a measurement collected from a single LG neuron from two different conditions: silent (gray) LG neurons where their active state was not induced or active (pink) LG neurons where their active state was induced for 8 h. The active state of LG neurons (pink) induced significant changes in ionic current magnitudes in 3/3 potassium currents (Welch's independent two-sample *t* test, *p* < 0.05) when compared with the silent condition. ***B***, Bar graphs (mean ± SD) displaying the corresponding mRNA abundances for the three potassium currents that were measured. Note that some ionic currents are encoded by more than one mRNA transcript. Each point corresponds to a measurement collected from a single LG neuron from two different conditions: silent (gray) LG neurons where their active state was not induced and active (pink) LG neurons where their active state was induced for 8 h. The active state of LG neurons (pink) induced significant changes in *BKKCA* (*I*_KCa_) and *SHAKER* (*I*_A_) when compared with the silent condition (Welch's independent two-sample *t* test, *p* < 0.05). ns, not significant. See also Extended Data Table 6-1. ***C***, Bar graphs displaying the change in the coefficient of variation (COV) for membrane currents (left panel) and ion channel mRNA abundances (right panel) during LG's silent and active state. The active state of LG neurons induced a significant decrease in the COV of all membrane currents but had no effect on the COV of mRNA abundances (Levene's test, *p* < 0.05 and *p* > 0.05, respectively). See also Extended Data Tables 2-1 and 2-2. * = *p* < 0.05, ** = *p* < 0.001, *** = *p* < 0.0001.

We also calculated coefficients of variation (COV) for ionic current magnitudes and for mRNA abundances of LG neurons (Fig. 2 and Extended Data Table 6-1). To test for significance between COVs, we used Levene's test. Changes in COV were considered significant if the *p*-value was <0.05.

10.1523/ENEURO.0320-24.2024.t2-1 Table 2-1 **LG ionic current peak magnitudes Pairwise T-test P-Values (Welch's independent two sample T-Test) between silent and active conditions.** Ionic current peak magnitudes pairwise comparisons between both groups. Download Table 2-1, DOCX file.

10.1523/ENEURO.0320-24.2024.t2-2 Table 2-2 **LG ion channel mRNA abundance Pairwise T-test P-Values (Welch**'s independent two sample T-Test) between silent and active conditions. mRNA abundance pairwise comparisons between both groups. Download Table 2-2, DOCX file.

## Results

### Activity induces changes in peak ionic current magnitudes and corresponding changes in ion channel mRNA abundances in LG neurons

We first determined if activating the gastric mill rhythm (Fig. 1*B*) would lead to changes in potassium currents (Fig. 1*C*) and in their corresponding ion channel mRNAs in LG cells compared with their silent state when the gastric mill is inactive (Fig. 1*B*). We predicted that turning on LG's active state for 8 h via *dpon* stimulation (Fig. 1*A*) would induce changes to LG's ion channel mRNA profile as previously reported (Viteri and Schulz, 2023).

Active LG neurons exhibited significantly different ionic current magnitudes for all three currents we measured (*I*_KCA_, *I*_A_, *I*_Kd_) compared with their silent counterparts (Fig. 2*A* and Extended Data Table 2-1). Specifically, the magnitude for *I*_KCa_ increased in active LG neurons (Welch's independent two-sample *t* test, *p* = 0.026) while the magnitudes for *I*_A_ and *I*_Kd_ decreased (Welch's independent two-sample *t* test, *p* = 0.037 and *p* = 0.026, respectively). Additionally, each ionic current is encoded by multiple distinct ion channel mRNAs (Fig. 1*C*). Active LG neurons exhibited significantly different changes in ion channel mRNA abundances for some of the mRNAs encoding the ionic currents we measured (Fig. 2*B* and Extended Data Table 2-2). Specifically, *BKKCA* (*I*_KCa_), and *SHAKER* (*I*_A_) all decreased significantly after 8 h of gastric mill rhythm activation (Welch's independent two-sample *t* test, *p* = 0.025, *p* = <0.001, and *p* = 0.017, respectively).

We also calculated the coefficient of variation (COV) for LG mRNA abundances and currents (Fig. 2*C*), to ascertain whether changes in activity and neuromodulation had an influence on the variability of both mRNAs and currents. We found that the active state of LG significantly reduced the COV of *I*_A_, *I*_KCa_, and *I*_Kd_ (Levene's test: *p* = 0.0063, *p* = 0.0201, and *p* = 0.0433, respectively) but had no effect on mRNA COVs.

### The interaction between ion channel mRNA correlations and ionic current correlations is cell type specific

Previous work has shown that ion channel mRNA pairwise relationships and their corresponding ionic current pairwise relationships can be similarly correlated (Schulz et al., 2006; Ransdell et al., 2012; Temporal et al., 2012). Therefore, we asked the following: if the active state of LG induces more ion channel mRNA relationships to become correlated (Viteri and Schulz, 2023), will it also induce the corresponding ionic currents to become more correlated?

**Figure 3.**
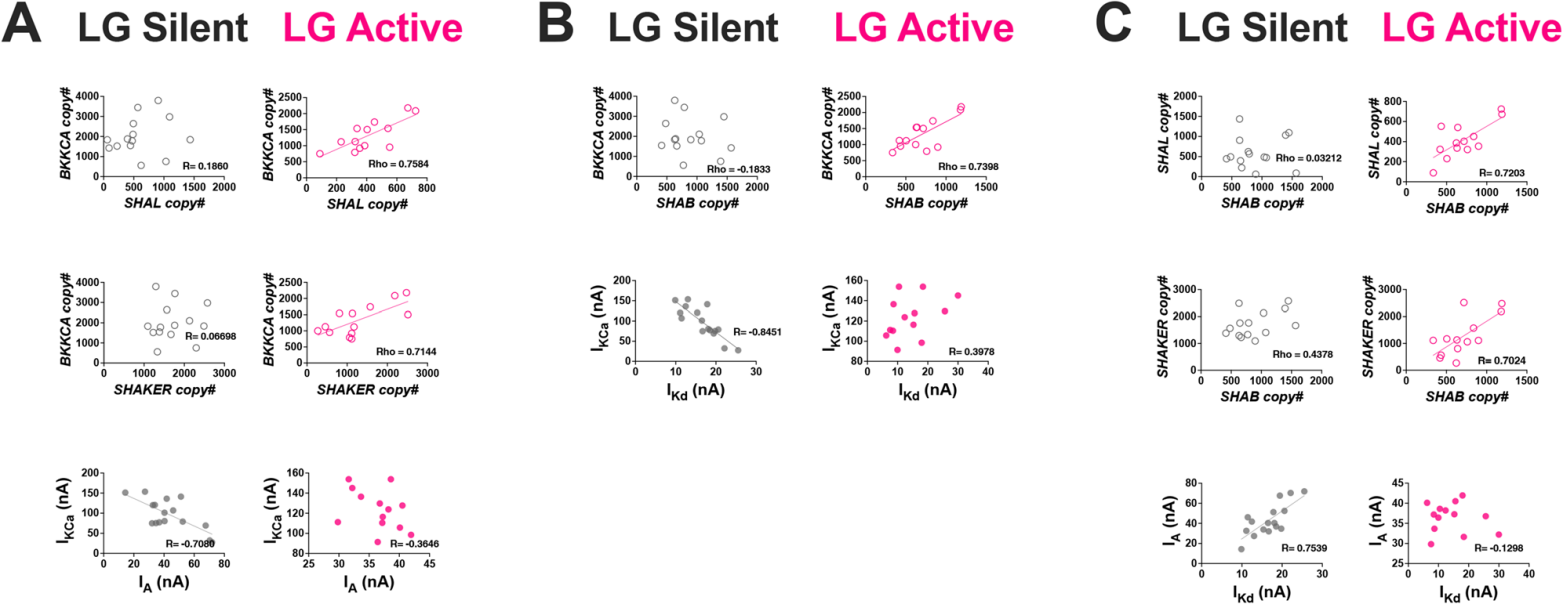
Ion channel mRNA relationships are correlated only in active LG neurons, but ionic current relationships are correlated only in silent LG neurons. ***A***, *BKKCA* versus *SHAL* and *BKKCA* versus *SHAKER* mRNAs were positively correlated only in active LG neurons (open pink circles: Spearman value > 0.6; *p* < 0.05). However, the corresponding ionic current relationship *I*_KCa_ versus *I*_A_ was negatively correlated only in silent LG neurons (solid gray circles: Pearson’s value < −0.6; *p* < 0.05). ***B***, *BKKCA* versus *SHAB* mRNAs were positively correlated only in active LG neurons (open pink circles: Spearman value > 0.6; *p* < 0.05). However, the corresponding ionic current relationship *I*_KCa_ versus *I*_Kd_ was negatively correlated only in silent LG neurons (solid gray circles: Pearson’s value greater than −0.6; *p* < 0.05). ***C***, The *SHAL* versus *SHAB* and *SHAKER* versus *SHAB* relationships were positively correlated in active LG neurons (open pink circles: Pearson’s value > 0.6; *p* < 0.05). However, the corresponding ionic current relationship *I*_A_ versus *I*_Kd_ was positively correlated only in silent LG neurons (solid gray circles: Pearson’s value > 0.6; *p* < 0.05). See also Extended Data Tables 3-1 and 3-2. *Pearson’s correlations denoted by “R.” *Spearman correlations denoted by “rho.”

We first quantified the pairwise relationship between *I*_KCa_ (*BKKCA*) and *I*_A_ (*SHAL/SHAKER*) in LG neurons. We found that the mRNA pairwise relationships *BKKCA* versus *SHAL* and *BKKCA* versus *SHAKER* were positively correlated in active but not silent LG neurons (Fig. 3*A* and Extended Data Table 4-1; Spearman value = 0.75 and 0.71, respectively; *p* = 0.003 and 0.006, respectively). To our surprise, the corresponding ionic current pairwise relationship *I*_KCa_ versus *I*_A_ was negatively correlated in silent but not in active LG neurons (Fig. 3*A* and Extended Data Table 4-2; Pearson’s value = −0.70, *p* = 0.002).

10.1523/ENEURO.0320-24.2024.t3-1 Table 3-1 **LG pairwise correlation values for ion channel mRNA relationships.** A relationship was considered to have become more correlated if the silent state R or Rho value was less than 0.6 (P-Value >0.05) and the active state R or Rho value was greater than 0.6 (P-Value <0.05). Download Table 3-1, DOCX file.

10.1523/ENEURO.0320-24.2024.t3-2 Table 3-2 **LG pairwise correlation values for ionic current relationships.** A relationship was considered to have become less correlated if the silent state R or Rho value was less than -0.6 (P-Value <0.05) or greater than 0.6 (P-Value <0.05) and the active state R or Rho value was less than 0.6 (P-Value >0.05) but greater than -0.6 (P-Value >0.05). Download Table 3-2, DOCX file.

Similarly, we quantified the pairwise relationship between *I*_KCa_ (*BKKCA*) and *I*_Kd_ (*SHAB*). The mRNA pairwise relationship *BKKCA* versus *SHAB* was positively correlated in active LG neurons (Fig. 3*B* and Extended Data Table 3-1; Spearman value = 0.74, *p* = 0.004). However, the corresponding ionic current pairwise relationship *I*_KCa_ versus *I*_Kd_ was negatively correlated in silent LG neurons but not in active LG neurons (Fig. 3*B* and Extended Data Table 3-2; Pearson’s value = −0.84, *p* < 0.0001).

Lastly, we quantified the pairwise relationship between *I*_A_ (*SHAL/SHAKER*) and *I*_Kd_ (*SHAB*). The mRNA pairwise relationships *SHAL* versus *SHAB* and *SHAKER* versus *SHAB* were positively correlated in active LG neurons (Fig. 3*C* and Extended Data Table 3-1; Pearson’s value = 0.72 and 0.7, respectively; *p* = 0.005 and 0.007, respectively), but the corresponding ionic current pairwise relationship *I*_A_ versus *I*_Kd_ was positively correlated in silent LG neurons silent but not in active LG neurons (Fig. 3*C* and Extended Data Table 3-2; Pearson’s value = 0.75, *p* < 0.0001).

**Figure 4.**
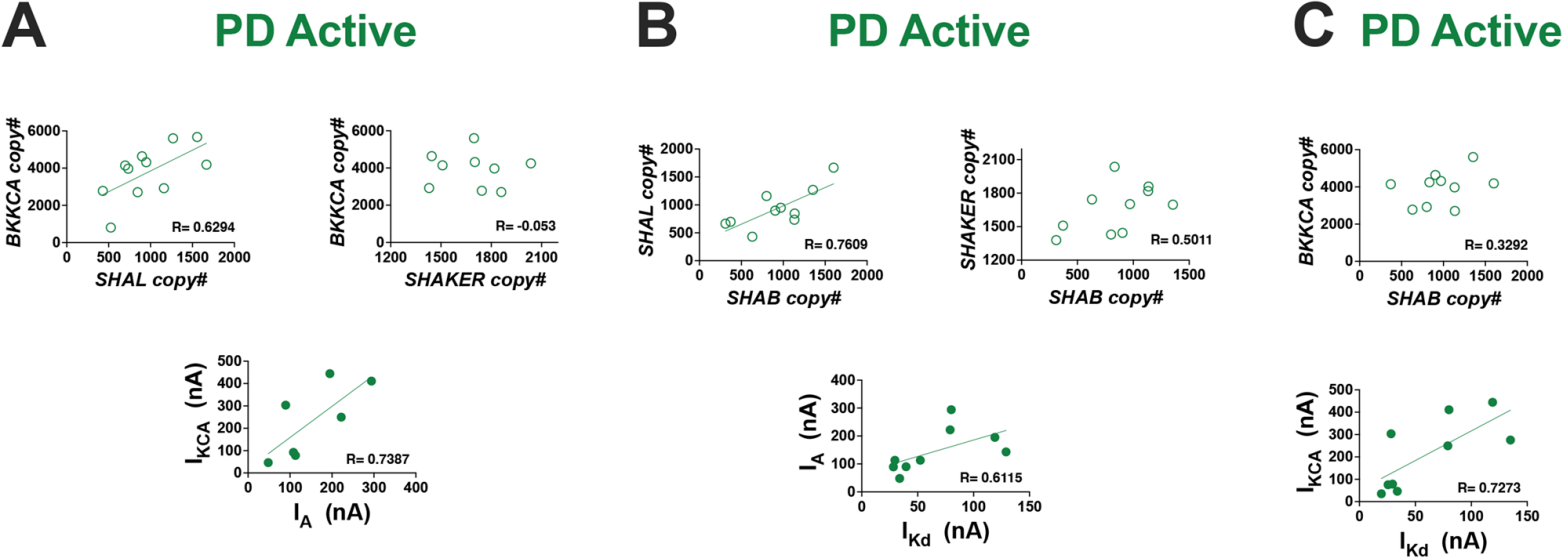
Both ion channel mRNA relationships and ionic current relationships are correlated in active PD neurons. ***A***, The *BKKCA* versus *SHAL* mRNA relationship was positively correlated (open pink circles: Pearson’s value > 0.6; *p* < 0.05) but not *BKKCA* versus *SHAKER* in active PD neurons. The corresponding ionic current relationship *I_KCa_* versus *I*_A_ was positively correlated (solid gray circles: Pearson’s value > 0.6; *p* < 0.05). ***B***, The *SHAL* versus *SHAB* mRNA relationship was positively correlated (open pink circles: Pearson’s value  > 0.6; *p* < 0.05) but not *BKKCA* versus *SHAB* in active PD neurons. The corresponding ionic current relationship *I*_A_ versus *I*_Kd_ was positively correlated (solid gray circles: Pearson’s value > 0.6; *p* < 0.05). ***C***, The *BKKCA* versus *SHAB* mRNA relationship was not correlated in active PD neurons. The corresponding ionic current relationship *I*_KCa_ versus *I*_Kd_ was positively correlated (solid gray circles: Pearson’s value > 0.6; *p* < 0.05). See also Extended Data Tables 4-1 and 4-2. *Pearson’s correlations denoted by “R.”

We then compared these results from LG neurons with similar data collected from active PD neurons. We first quantified the pairwise relationship between *I*_KCa_ (*BKKCA*) and *I*_A_ (*SHAL/SHAKER*). We found that the mRNA pairwise relationship *BKKCA* versus *SHAL* was positively correlated but *BKKCA* versus *SHAKER* was not correlated (Fig. 4*A* and Extended Data Table 4-1; Pearson’s value = 0.63 and −0.05, respectively; *p* = 0.038 and 0.891, respectively). The corresponding ionic current pairwise relationship *I*_KCa_ versus *I*_A_ was positively correlated in PD neurons (Fig. 4*A* and Extended Data Table 4-2; Pearson’s value = 0.74, *p* = 0.058).

10.1523/ENEURO.0320-24.2024.t4-1 Table 4-1 PD pairwise correlation values for ion channel mRNA relationships. Download Table 4-1, DOCX file.

10.1523/ENEURO.0320-24.2024.t4-2 Table 4-2 PD pairwise correlation values for ionic current relationships. Download Table 4-2, DOCX file.

Similarly, we quantified the pairwise relationship between *I*_A_ (*SHAL/SHAKER*) and *I*_Kd_ (*SHAB*). The mRNA pairwise relationship *SHAL* versus *SHAB* was positively correlated but *SHAKER* versus *SHAB* was not correlated (Fig. 4*B* and Extended Data Table 4-1; Pearson’s value = 0.761 and 0.501, respectively; *p* = 0.011 and 0.141, respectively). The corresponding ionic current pairwise relationship *I*_A_ versus *I*_Kd_ was positively correlated (Fig. 4*B* and Extended Data Table 4-2; Pearson’s value = 0.612, *p* = 0.011).

Lastly, we quantified the pairwise relationship between *I*_KCa_ (*BKKCA*) and *I*_Kd_ (*SHAB*). The mRNA pairwise relationship *BKKCA* versus *SHAB* was not correlated in PD neurons (Fig. 4*C* and Extended Data Table 4-1; Pearson’s value = 0.329, *p* = 0.326). However, the corresponding ionic current pairwise relationship *I*_KCa_ versus *I*_Kd_ was positively correlated (Fig. 4*C* and Extended Data Table 4-2; Pearson’s value = 0.727, *p* = 0.026).

### Ion channel mRNA abundances are directly correlated with their ionic currents in PD neurons, but not in LG neurons

**Figure 5.**
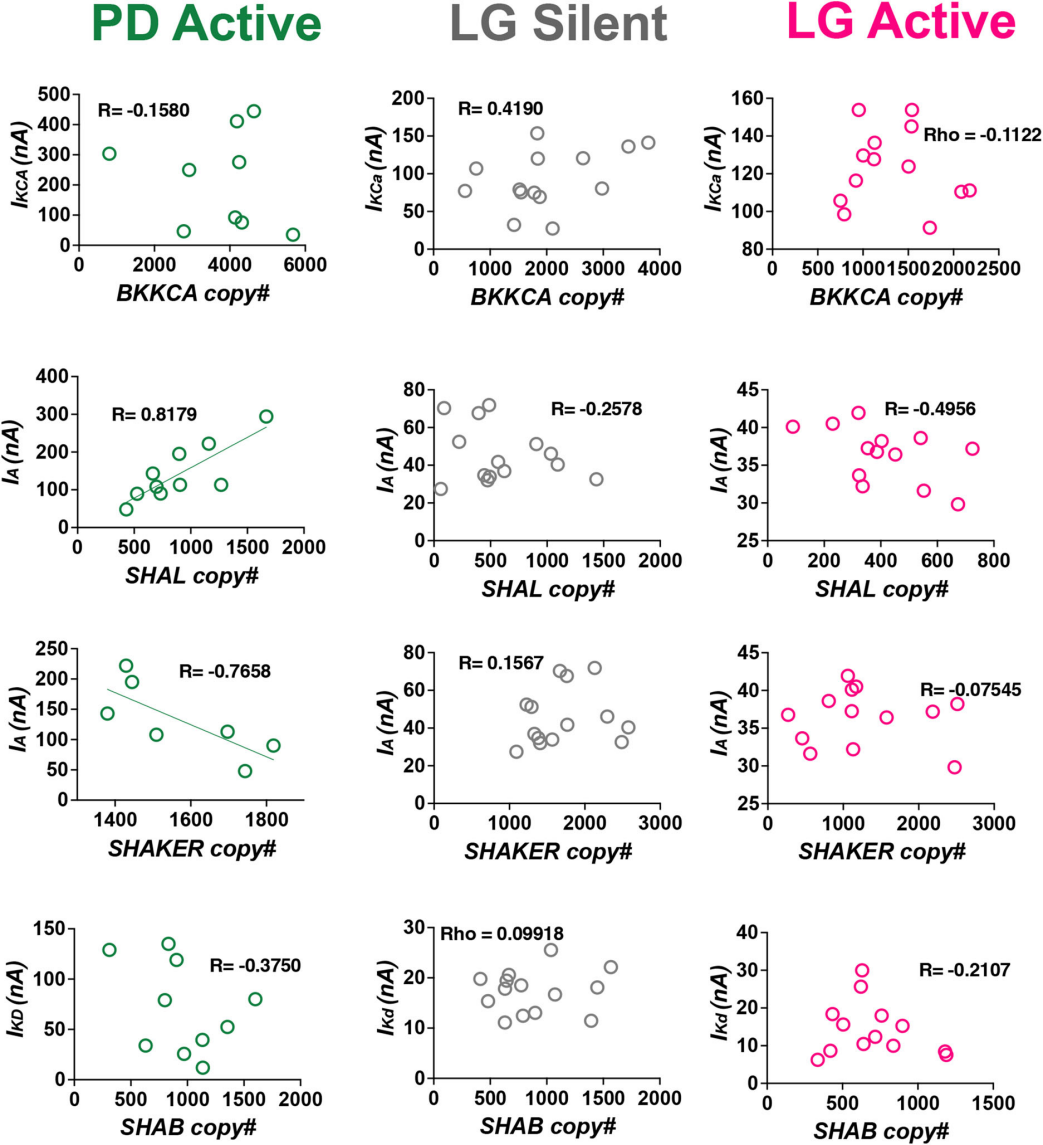
Ion channel mRNA transcripts are correlated with the ionic currents they encode in PD, but not LG neurons. Ion channel mRNAs were plotted against the ionic currents they encode across PD and LG neurons (silent and active state). The *I*_A_ versus *SHAL* relationship is positively correlated in PD (open green circles: Pearson’s value > 0.6; *p* < 0.05). The *I*_A_ versus *SHAKER* relationship is negatively correlated in PD (open green circles: Pearson’s value > 0.6; *p* < 0.05). However, none of these relationships were correlated in silent or active LG neurons (open gray and pink circles). See also Extended Data Table 5-1. *Pearson’s correlations denoted by “R.”

If continually active PD neurons use activity-dependent feedback to maintain both correlated channel mRNA relationships and ionic current relationships (Temporal et al., 2012; Santin and Schulz, 2019), then we predicted that there should be a direct correlation between a given ionic current and the underlying channel mRNAs that encode it in PD. In support of this, previous work has shown that in another continually active neuron of the STG, the lateral pyloric neuron (LP), some channel mRNAs are directly correlated with the ionic current they encode (Schulz et al., 2006). Conversely, since LG is able to restart its normal output after periods of inactivity, the ionic current profile necessary to do so should already be established in LG's inactive state. Thus, since we have previously reported that many new channel mRNA correlations are formed during LG's active state but not during its silent state (Viteri and Schulz, 2023), we also predicted that we would not observe any individual mRNAs correlated with their corresponding ionic currents in LG neurons of either state (silent or active). To test this, we looked for correlations among each ionic current we measured against the channel mRNAs that encode it in active PD neurons as well as in both silent and active LG neurons. We found that in active PD neurons, *I*_A_ versus *SHAL* was positively correlated, and *I*_A_ versus *SHAKER* was negatively correlated (Fig. 5 and Extended Data Table 5-1; Pearson’s value = 0.82 and −0.77, respectively; *p* = 0.004 and 0.044, respectively). Conversely, in neither silent nor active LG neurons did we find correlated ion channel mRNAs versus their corresponding ionic currents (Fig. 5 and Extended Data Table 5-1; all absolute Pearson’s correlations values were <0.6 and *p* > 0 0.05).

10.1523/ENEURO.0320-24.2024.t5-1 Table 5-1 Pairwise correlation values for ionic currents vs mRNA relationships. Download Table 5-1, DOCX file.

## Discussion

**Figure 6.**
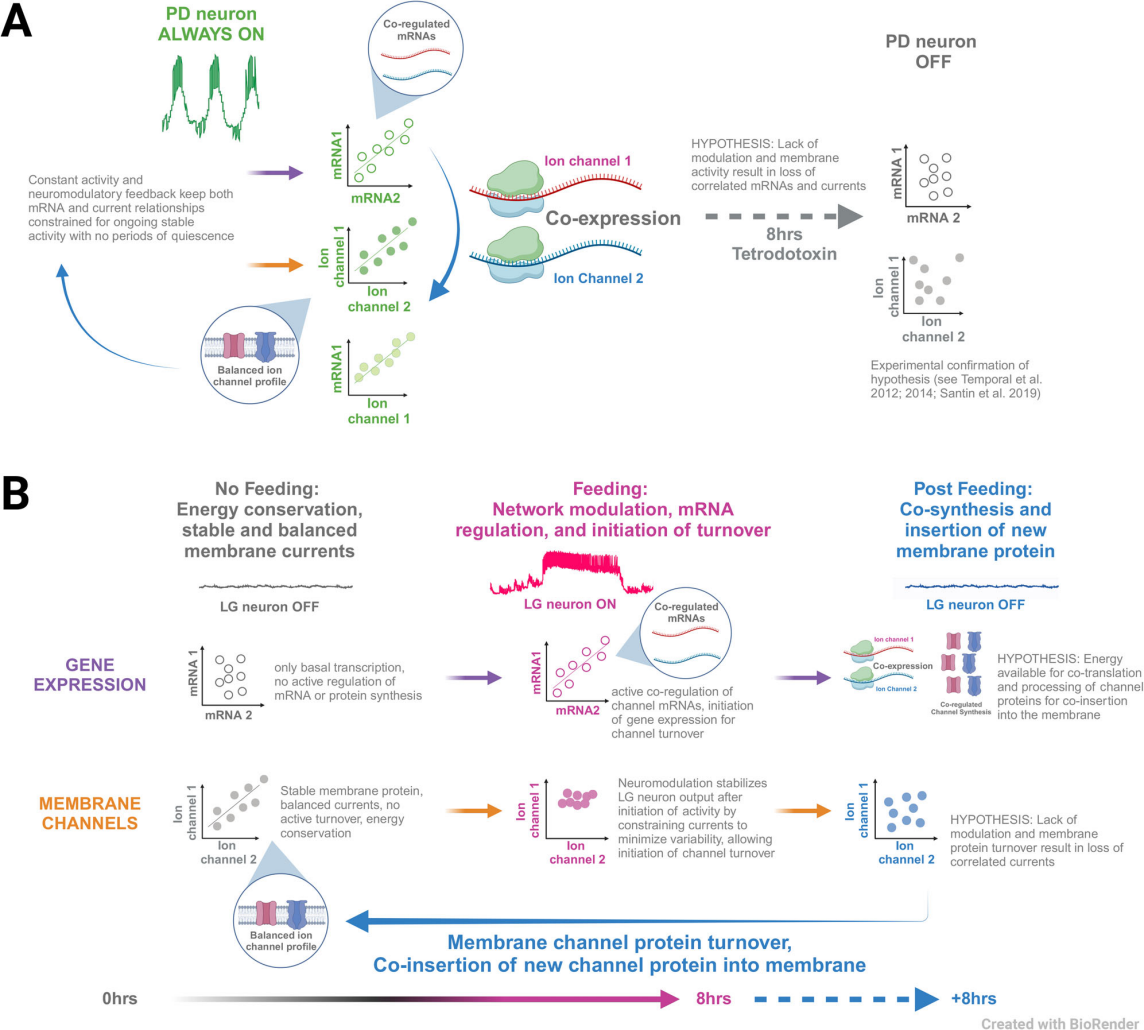
A model for temporally uncoupled regulation of channel mRNA and protein in episodically versus continually active neurons. ***A***, In PD neurons (continually active), there is continuous activity- and modulator-dependent feedback that signals to maintain and tune both channel mRNA and protein relationships in a continually coregulated state, so that ongoing activity can continue throughout the lifetime of the animal without interruption. This ongoing regulation is revealed by artificially silencing PD neurons with tetrodotoxin (TTX): when PD neurons are experimentally turned OFF (silent), both mRNA and membrane current relationships that are correlated in the active state are no longer maintained (Temporal et al., 2012, 2014; Santin and Schulz, 2019). ***B***, In LG neurons (episodically active), when the animal is (1) in the “No Feeding” state, the gastric mill is silent, and the LG neuron is in its OFF state with no activity. In this state, membrane currents are correlated and presumably balanced to generate appropriate cell type–specific output on demand. Concurrently, the mRNA relationships for these channels are not actively being maintained (hence not correlated). When (2) “Feeding” is initiated, the gastric mill—including LG—becomes active. This results in LG neurons receiving both activity- and modulator-dependent feedback. Our data indicate that these feedback pathways result in coregulated channel mRNAs, manifesting as correlated channel mRNA abundance (Viteri and Schulz, 2023). Meanwhile, measured membrane currents are no longer correlated. However, the variability of the magnitudes of these currents across individuals is significantly decreased during the active state of LG (Fig. 2*C*), suggesting that neuromodulation influences state-dependent relationships among these currents to ensure robust output (Marder and Bucher, 2007; Stein, 2009) In the (3) “Post Feeding” phase, we hypothesize that the correlated mRNAs are used as templates for coregulated translation and processing of ion channel proteins, which are then turned over in the membrane (solid blue arrow) to prepare the LG neurons for the next feeding cycle. These new channels ensure appropriate output is generated again on demand, tuned by the feedback received in the previous activity cycle.

In this study, we investigated how motor neurons with different activity profiles (continually vs episodically active) coordinate ion channel mRNA and ionic current relationships. We found that PD and LG neurons of the crustacean STG manifest correlated ion channel mRNAs and correlated ionic currents differently: PD neurons (continually active) exhibit temporally overlapping mRNA and current relationships (Fig. 4), while in LG neurons (episodically active), the presence of mRNA and current correlations is distinct (Fig. 3) across silent and active states. This leads to a fundamentally different relationship between channel mRNAs and the currents they encode in these cell types: in PD neurons, channel mRNAs and their resulting currents are directly (simultaneously) correlated when measured in the same neurons while no such relationships are evident in LG neurons. We have summarized our major findings in a proposed model in Figure 6, which serves as a framework to interpret our results and for future work.

10.1523/ENEURO.0320-24.2024.t6-1 Table 6-1 **LG coefficient of variation and Levene's test.** COV was calculated for every ionic current measured, and Levene's test p-values were computed to ascertain if variation difference between conditions were significantly different. Download Table 6-1, DOCX file.

The balance of different ionic currents determines the electrical output of a neuron. However, the magnitude of individual ionic currents can vary markedly across neurons of the same population (Prinz et al., 2004; Schulz et al., 2006). One possible strategy to reconcile this population variability with each neuron's need to produce reliable and robust patterned activity is to coregulate levels of ionic current expression (Tran et al., 2019). In continuous rhythmically active motor neurons of the crustacean cardiac ganglion, for example, blocking the high threshold potassium current alters the ongoing activity of a motor neuron, but within 1 h, the A-type potassium current increases and restores that neuron's activity (Ransdell et al., 2012). This demonstrates the coregulation of these currents and suggests that correlations among currents or their channel mRNAs (Schulz et al., 2007) contribute to maintaining robust activity. Such coregulation is manifested in PD neurons of the STG, where in intact systems with continuous activity, both membrane currents and the channel mRNAs that encode them are correlated with one another (Fig. 6*A*, PD always on; see also Temporal et al., 2012). Furthermore, when activity in these PD neurons is artificially stopped, both membrane current and channel mRNA correlations are disrupted (Fig. 6*A*, 8 h tetrodotoxin; see also Temporal et al., 2012, 2014; Santin and Schulz 2019). Membrane voltage has been demonstrated to be the signal maintaining many of these mRNA relationships in PD neurons (Santin and Schulz, 2019), with evidence also suggesting the role of neuromodulatory feedback in maintaining these relationships in PD (Temporal et al., 2012, 2014; Santin and Schulz, 2019). Taken together, these studies suggest that activity-dependent feedback maintains the coregulation of both ionic conductances and the channel mRNAs that encode them, ultimately resulting in the stabilization of ongoing activity in rhythmically active motor neurons. However, given that all these lines of evidence were obtained from continually active neuron types, this raises the question: how do neurons that possess only episodic patterns of activity establish the necessary balance of ionic currents to resume their activity after periods of extended quiescence?

Unlike continually active neurons of the pyloric network, LG cells possess two natural states (Fig. 6*B*): a lengthy silent state when the animals are not feeding and an episodically active state when food is consumed (Cook and Nusbaum, 2021). Despite experiencing extended periods of silence, the gastric mill (including LG) is able to immediately resume normal activity upon stimulation with food (Clemens et al., 1998; McGaw and Curtis, 2013) or hemolymph from animals that have recently fed (Cook and Nusbaum, 2021; DeLaney et al., 2022) or by artificially activating descending neuromodulatory inputs (Fig. 6*B*; Beenhakker et al., 2004), presumably because LG possesses the appropriate profile of ionic currents at the time of activation. By turning on LG's normal activity in vitro for an 8 h period (a period approximating regular feeding time in live animals; Cook and Nusbaum, 2021), we found that some channel mRNA abundances changed, although interestingly, some in the opposite direction as the ionic current they encode (Fig. 2). We previously reported that after 8 h of gastric mill activity, 7/11 measured mRNA transcripts increased in LG neurons, including *SHAKER*, *SHAB*, and *BKKCA* (Viteri and Schulz, 2023). In this study, we report that these same mRNA transcripts decrease or are not changed in abundance after gastric mill activity (Fig. 2). What is consistent across studies is that after 8 h of gastric mill activity, the mRNA pairwise relationships in this active state for LG neurons that we report here (Fig. 3) are the same relationships we found in our previous study in LG neurons (Viteri and Schulz, 2023). This suggests that ion channel mRNA pairwise relationships may be a more stable marker of the presence of activity- and cell type–dependent regulatory programs necessary for appropriate neuronal outputs than simply the abundance of any given channel mRNA. For the membrane currents encoded by these channel proteins, we also measured changes in magnitude associated with the activation of LG neurons: *I*_KCa_ increased and *I*_Kd_ and *I*_A_ decreased significantly after 8 h of gastric mill activity (Fig. 2). Like our results for channel mRNA abundance, correlations among these membrane currents also changed as a result of LG activity. However, to our surprise, the membrane currents in LG were correlated only in the silent state and not after 8 h of activity (Fig. 3), while the opposite held true for mRNA relationships. This is unlike PD neurons that clearly show simultaneous coregulation of both membrane current relationships and channel mRNA abundances during their active state (Fig. 4).

Taken together, we propose that this lag between the regulation of relationships among channel mRNAs and their resulting ionic currents in episodically active neurons such as LG may be a mechanism to ensure that robust activation of appropriate output remains possible in the next bout of feeding, which may be days or even weeks subsequent to the event that triggers this regulatory cascade (Scharf, 2016). We propose a working model (Fig. 6) based on the need to balance the protein turnover of ion channels with the unpredictable nature of feeding and energetics in this circuit. The major features of this model are as follows.

First, our data indicate that in a quiescent gastric mill network (Fig. 6*B*, “no feeding”), channel mRNAs are present in LG but uncorrelated while membrane currents show clear pairwise relationships. We suggest that in this state, membrane channel proteins are stable and balanced (Khorkova and Golowasch, 2007; Zhao and Golowasch 2012) to immediately allow for the generation of cell type–specific output upon activation of the gastric mill. We further propose that in this state, while baseline transcription of channel mRNAs remains active, subsequent post-transcriptional processing and translation of channel mRNAs have been shut down to minimize energy expenditure for what can be quite extended periods without feeding (Scharf, 2016). While transcription does have an energetic cost (Cheng et al., 2019; Laloum and Robinson-Rechavi, 2022), the most energetically expensive aspects of gene expression are post-transcriptional and translational processes, which are estimated to have on the order of 100× more energetic cost than transcription (Lynch, 2024). Hence, by suspending the organization of mRNA relationships and the production of new protein, we speculate that the animal can help minimize energy expenditure during times when food is not available.

Second, activation of the gastric mill occurs as a result of feeding (Fig. 6*B*, “feeding”) and most proximally the release of neurotransmitters and neuropeptide modulators into the circuit (DeLong et al., 2009). We propose that the instantaneous generation of appropriate output by LG is due to the presence of existing, stable channel protein relationships in the membrane (as measured in the quiescent state) that result in ionic currents being balanced in generating output. While neuromodulation is necessary for the activation of the gastric mill, the effects of most neuropeptides reach their peak relatively slowly and are long-lasting modulators of behavior (Merighi et al., 2011; Flavell et al., 2013). In LG neurons, peptide modulators have multiple targets, including the activation of a driving current known as the “mixed-inward” current (*I*_MI_) as well as synapses in the circuit (Norris et al., 1994; Stein et al., 2007; Blitz et al., 2008; DeLong et al., 2009). We suggest the existing relationships among membrane currents in the quiescent state of LG balance with an initial excitatory drive from descending projections in the initial cycles of LG activity to ensure robust output immediately upon activation. However, as activity persists and the slower effects of peptide modulation alter the circuit, a new solution or parameter space for LG is reached—in part by alteration of membrane conductances such as *I*_A_, *I*_KCa_, and *I*_Kd_—to optimize and stabilize output for what can be several hours of circuit activity. These changes are manifested both as a loss of correlation (Fig. 3) as well as a decrease in the coefficient of variation (Fig. 2*C*) among these currents relative to the quiescent state and may indicate that these currents are altered or modulated to balance other now-present conductances such as *I*_MI_ or calcium-dependent currents. This decrease in variability may reflect a smaller range of conductances such as *I*_MI_ in LG after extended gastric activity, although this smaller variation of *I*_MI_ would not be consistent with what has been reported after treatment with the neuropeptide proctolin in the continually active LP neurons of the pyloric circuit (Schneider et al., 2022). Furthermore, these peptide modulators are known to alter synaptic properties within the gastric circuit (Blitz and Nusbaum, 2012; Fahoum and Blitz, 2024), and changes in membrane conductance may subsequently be needed to balance changes in synaptic dynamics among gastric neurons. Hence, these relationships present in the quiescent state are no longer maintained and/or necessary for appropriate output, and correlations are no longer detectable among them.

Third, in parallel with the membrane conductance changes described above, we propose that gene expression in LG is also regulated by gastric mill activation (Viteri and Schulz, 2023). Specifically, during gastric activity either baseline transcription, post-transcriptional regulation of mRNA abundance (Bhat et al., 2022), or both, is altered to coregulate levels of channel mRNAs so that coexpression can result in balanced ion channel protein profiles for future activation. Furthermore, correlated mRNA levels may represent organization into structures such as neuronal granules (Kiebler and Bassell, 2006) that can be trafficked to the appropriate neuronal compartment, and thus translation can be silenced in the cytoplasm until local translation can occur in this coordinated fashion (Formicola et al., 2019; Oliveira and Klann, 2021). Ultimately, we suggest that the production of these channel proteins—whether they are translated locally or nonspecifically in the soma—is triggered by LG activation and these channels are coinserted in the membrane during protein turnover in the periods between feeding bouts (Fig. 6*B*, “postfeeding”), restoring the correlated ionic current profiles that we measured in the LG quiescent state.

Lastly, previous work in LP neurons of the STG (Schulz et al., 2006) and in dopaminergic neurons of the substantia nigra (Liss et al., 2001) showed a strong relationship between membrane current for *I*_A_ and *I*_KCa_ and mRNA copy number for the channels that encode them. When we measured mRNAs and currents from the same PD neurons, we found yet another clear example of a direct correlation between channel mRNA (*SHAL*) and A-type current magnitude (Fig. 5). When making these same comparisons in silent and active LG neurons, we found that none of the currents measured were correlated with the mRNAs that encode them (Fig. 5). What is striking about these results is that for all three of the examples where channel mRNAs and currents strongly correlate to one another in single cells (Liss et al., 2001; Schulz et al., 2006), not only is the current involved the A-type potassium current, but all three of these cell types are continually active. Conversely, in the one example of an episodically active neuron—the LG data in this study—these relationships were not detected. This is consistent with our hypotheses that continually and episodically active cells have distinct mechanisms to maintain their properties over time, and due to the temporal uncoupling of channel mRNA and current in episodically active cells, we would not expect to see a direct relationship between the two.

Admittedly, much of the model proposed above is speculative, albeit consistent with the data at hand. Our model makes a clear distinction in mechanisms constraining channel mRNAs and membrane currents between episodically active neurons like LG (Fig. 6*B*) and continually active neurons like PD (Fig. 6*A*). This working model also allows us to propose several testable hypotheses. For example, if the loss of correlations among membrane currents after gastric mill activity is indicative of a rebalancing of these conductances with *I*_MI_, then if we were to simultaneously measure K^+^ currents and *I*_MI_ in LG after gastric activity, we might expect a correlation among some subset of these currents with *I*_MI_. Second, after ceasing the activation of modulatory inputs, but prior to the completion of membrane channel turnover proposed during the interbout interval, we predict that potassium currents would once again be more variable and uncorrelated (Fig. 6*B*), resulting in subsequent attempts to activate the gastric mill that fail or result in lack of or altered output of LG relative to the normal patterned activity. Indeed, anecdotally this latter effect has been observed in our preparations—with the substantial caveat that we believe that extended electrical stimulation of the *dpon* nerves likely leads to damage and/or “burning out” of these inputs and hence a major confound in these observations. Further work is clearly needed to put this new model through its paces to determine where it may capture some of these network dynamics appropriately.

## Synthesis

Reviewing Editor: David Schoppik, New York University Grossman School of Medicine Decisions are customarily a result of the Reviewing Editor and the peer reviewers coming together and discussing their recommendations until a consensus is reached. When revisions are invited, a fact-based synthesis statement explaining their decision and outlining what is needed to prepare a revision will be listed below. The following reviewer(s) agreed to reveal their identity: NONE. Note: If this manuscript was transferred from JNeurosci and a decision was made to accept the manuscript without peer review, a brief statement to this effect will instead be what is listed below.

1. The literature about CPG divided rhythms into continuous vs episodic. We are all in agreement that the manuscript would be considerably stronger if it used "episodic" rather than "periodic."

Examples where episodic fits better: "This may ensure robust activation of appropriate output is possible in the next bout of feeding, which may be days or even weeks subsequent to the event that triggers this regulatory cascade (Scharf I 445 2016)..." "...temporal uncoupling of this process may occur in periodically active cells like LG; whereby feedback during one cycle of activity..." LG has a burst cycle during it episodic activation, which is event driven.

2. Please make it clear that R is for Pearson and Rho is for Spearman in the Figure Legends.

3. We felt that the final figure, and how it is used in the text, could use work: as it stands, it is not explanatory or otherwise helpful. We realize it's a proposed model and speculative, but while speculating, it would be nice to include the author's thoughts on the changes in the ionic currents during the 8 hour period. If the currents are set up to be ready for the next active period, why do they change during activity? Why aren't they in the perfect state and just stay that way during activity? Does activity drive these changes in order to move the cell back to its silent state? Does it have to do with the timing of membrane protein turnover? The manuscript would benefit from a supported or at least novel hypothesis about why these changes occur to fill in that gap in the model of a temporal mismatch?

4. The Discussion would benefit from pointing out the confounds in the data.

5. Eight hours is a long episode of activity, and it is unclear how long is needed for the episode driven changes to occur. Let's say they are present after four hours, why aren't these mRNA changes expressed during the activity only the quiescent channel expression is maintained? How long must the quiescent period be to bring about the quiescent changes. Why does the cell even bother to change during an episode if it is preadapted during quiescence? The manuscript states that "Given the energetic costs of transcription (Laloum D and Robinson-rechavi M 2022; Cheng Y et al. 2019) and the infrequency with which crustaceans feed (Scharf I 2016), maintaining ion channel mRNAs throughout LG's inactive state could be energetically inefficient." , but we do not see a tremendous reduction in mRNA levels during the quiescent state -- rather the opposite.

6. There is an apparent mismatch between some of the data and how it is discussed in the results and discussion sections. There does not appear to be any difference in SHAB levels, yet there is more than 1 location in which the authors indicate that at least one mRNA transcript for each current, or all measured mRNAs were different between silent and active LGs. But that does not seem to be true for Ikd/SHAB.

Please update the references: there are instances of many authors instead of "et al" and references within the text include authors' first initials. There are also issues with the reference list including errors in the Schulz et al 2006 and following 2007 references and journal names are not abbreviated.

• Lines 119, 204, 207, use degree symbol

• Line 122, If the british spelling "oesophageal" is used in this system, the abbreviation OG makes sense, and lower case italicized nerve names are standard in this system. Please fix throughout text.

• Lines 129, 148 (2x), 149, 179 (2x), etc., please insert "mill" after gastric when referring to gastric mill activity or the gastric mill rhythm etc.

• Line 149 to 150, a rhythm doesn't burst, neurons burst

• Line 232, change of to or

• Line 233, change neurons to neuron

• Line 297, the data do not seem to support this statement "Active LG neurons exhibited significantly different ion channel mRNA abundance for at least one of the mRNAs that encodes each ionic current". It seems that there is no significant abundance change for IKD (shal) mRNA. (also referred to in line 428).

• Line 330, delete the second "silent"

• Line 374. Why isn't any of the data referred to for this final result?

• Discuss model within discussion? That one sentence is confusing.

• Line 429, "all channel mRNAs we measured decreased significantly after 8 hours of gastric activity (Figure 2)." But Figure 2 and the figure 2 legend, indicate that Shab and Shal did not change significantly. Is this an error in labeling or in description in the text? There does seem to be a trend, but not a statistical difference?

• Line 542, insert "the" before 3

• Line 603, why isn't modulator-dependent feedback discussed in the discussion as one of the differences between PD and LG? Again, the model is discussed at all, just a brief mention that there is one in the figure.

• Figure 6B. Why is modulator-dependent feedback indicated for the active PD? Viteri and Schulz 2023 is cited, which demonstrated modulator and activity-dependent feedback for LG but perhaps there were earlier studies that also showed some modulator-dependent feedback in PD?

• Line 608, The information is Table 6-1 is not mentioned in the results, We only see it in the figure legend for figure 6, which is only mentioned in the discussion.

• Figure 6. A vertical format might work better so that the two pieces could be enlarged. The text and schematics seem small in the horizontal layout.
