# Supplementary material for: Distinct Strategies Regulate Correlated Ion Channel mRNAs and Ionic Currents in Continually versus Episodically Active Neurons
Source: eNeuro. 2024 Nov 12;11(11):ENEURO.0320-24.2024. doi: 10.1523/ENEURO.0320-24.2024 (PMC11574698; doi:10.1523/ENEURO.0320-24.2024)
Supplement: Table 1-1 — Primers and probes used for multiplex RT-QPCR reactions used in LG neurons. Download Table 1-1, DOCX file. [file eneuro-11-ENEURO.0320-24.2024-s002.docx]

**Supplementary Tables**

| Gene | Current/Protein | Forward/Reverse Primer 5’-3’ Sequence | Probe 5’-3’ Sequence |
| --- | --- | --- | --- |
| *SHAL* | I_A_ | GACACCACCTTCACCTCCATTC / GAACCATGTCGCCGTATCCTA | CGGCGTTTTGGTACACCATTGTCAC |
| *SHAKER* | I_A_ | GAGGCTCAGAAGACCAGTCAAC / TGGCGATATCACCGAGCTCAT | CACTCGATGTCTTCGCGGAGGAGAT |
| *SHAB* | I_Kd_ | GAGCCGGACAGACAGGAAC / TGCGCCTCCTTCTGTAGTC | AAGAACCACGAACACCACATGGGTC |
| *BKKCA* | I_KCa_ | GCTCAAACTCGGCTTCATTG / CTGCGTGTCTGGAGAAGTTT | AGAATCCCGGCGCTAAACATGACT |
| *VLGUT* | Glutamate Transporter | GCGTTCGTGGACCTTCTAC / TCAGCCACCCTGTAATGGAA | TCAGCCACCCTGTAATGGAA |
| *CHAT* | Choline Acetyl-transferase | GGACCGCCTGGCTAAGTAC / TCGCGGAGTCCCATAAGG | AGGCGGCGCTCAAGCTTCAGAC |
| *VACHT* | Vesicular Acetyl-choline transporter | GCGTCAGCTGCTTCTTCCT / CAGCAGTGCCGTGTCTATGAG | TTCGCCAGCAACTACTGGGTGTT |
| *ACHE* | Acetylcholin-esterase | GGGCAACATGGGCATGTAC / GGTCACCACCGAAGAATTCAATG | AGGCGCTGGCCATCAAGTGGATAC |

**Table 1-1. Primers and probes used for multiplex RT-QPCR reactions used in LG neurons.**
