# Supplementary material for: Distinct Strategies Regulate Correlated Ion Channel mRNAs and Ionic Currents in Continually versus Episodically Active Neurons
Source: eNeuro. 2024 Nov 12;11(11):ENEURO.0320-24.2024. doi: 10.1523/ENEURO.0320-24.2024 (PMC11574698; doi:10.1523/ENEURO.0320-24.2024)
Supplement: Table 2-1 — LG ionic current peak magnitudes Pairwise T-test P-Values (Welch's independent two sample T-Test) between silent and active conditions. Ionic current peak magnitudes pairwise comparisons between both groups. Download Table 2-1, DOCX file. [file eneuro-11-ENEURO.0320-24.2024-s003.docx]

| **Current** | **Silent v Active** |
| --- | --- |
| I_A_ | 0.0372 |
| I_KCa_ | 0.0256 |
| I_Kd_ | 0.0257 |

**Table 2-1. LG ionic current peak magnitudes Pairwise T-test P-Values (Welch’s independent two sample T-Test) between silent and active conditions.** Ionic current peak magnitudes pairwise comparisons between both groups.
