# Supplementary material for: Distinct Strategies Regulate Correlated Ion Channel mRNAs and Ionic Currents in Continually versus Episodically Active Neurons
Source: eNeuro. 2024 Nov 12;11(11):ENEURO.0320-24.2024. doi: 10.1523/ENEURO.0320-24.2024 (PMC11574698; doi:10.1523/ENEURO.0320-24.2024)
Supplement: Table 2-2 — LG ion channel mRNA abundance Pairwise T-test P-Values (Welch's independent two sample T-Test) between silent and active conditions. mRNA abundance pairwise comparisons between both groups. Download Table 2-2, DOCX file. [file eneuro-11-ENEURO.0320-24.2024-s004.docx]

| **Gene** | **Silent v Active** |
| --- | --- |
| *SHAB* | 0.1526 |
| *SHAKER* | 0.0007 |
| *SHAL* | 0.1373 |
| *BKKCA* | 0.0253 |

**Table 2-2. LG ion channel mRNA abundance Pairwise T-test P-Values (Welch’s independent two sample T-Test) between silent and active conditions.** mRNA abundance pairwise comparisons between both groups.
