# Supplementary material for: Distinct Strategies Regulate Correlated Ion Channel mRNAs and Ionic Currents in Continually versus Episodically Active Neurons
Source: eNeuro. 2024 Nov 12;11(11):ENEURO.0320-24.2024. doi: 10.1523/ENEURO.0320-24.2024 (PMC11574698; doi:10.1523/ENEURO.0320-24.2024)
Supplement: Table 3-1 — LG pairwise correlation values for ion channel mRNA relationships. A relationship was considered to have become more correlated if the silent state R or Rho value was less than 0.6 (P-Value >0.05) and the active state R or Rho value was greater than 0.6 (P-Value <0.05). Download Table 3-1, DOCX file. [file eneuro-11-ENEURO.0320-24.2024-s005.docx]

| **Relationship** | **Silent Correlation Value (Pearson R or Spearman Rho)** | **P-Value** | **Active Correlation Value (Pearson R or Spearman Rho)** | | **P-Value** |
| --- | --- | --- | --- | --- | --- |
| *BKKCA* v *SHAL* | R = 0.1860 | 0.5248 | Rho = 0.7584 | 0.0027 | |
| *BKKCA* v *SHAKER* | R = 0.0669 | 0.8199 | Rho = 0.7144 | 0.0061 | |
| *BKKCA* v *SHAB* | Rho = -0.1833 | 0.5299 | Rho = 0.7398 | 0.0038 | |
| *SHAL* v *SHAB* | Rho = 0.0321 | 0.9137 | R = 0.7203 | 0.0055 | |
| *SHAKER* v *SHAB* | Rho = 0.4378 | 0.1175 | R = 0.7024 | 0.0074 | |
|  |  |  |  |  | |

**Table 3-1. LG pairwise correlation values for ion channel mRNA relationships.** A relationship was considered to have become more correlated if the silent state R or Rho value was less than 0.6 (P-Value >0.05) and the active state R or Rho value was greater than 0.6 (P-Value <0.05).
