# Supplementary material for: Distinct Strategies Regulate Correlated Ion Channel mRNAs and Ionic Currents in Continually versus Episodically Active Neurons
Source: eNeuro. 2024 Nov 12;11(11):ENEURO.0320-24.2024. doi: 10.1523/ENEURO.0320-24.2024 (PMC11574698; doi:10.1523/ENEURO.0320-24.2024)
Supplement: Table 3-2 — LG pairwise correlation values for ionic current relationships. A relationship was considered to have become less correlated if the silent state R or Rho value was less than -0.6 (P-Value <0.05) or greater than 0.6 (P-Value <0.05) and the active state R or Rho value was less than 0.6 (P-Value >0.05) but greater than -0.6 (P-Value >0.05). Download Table 3-2, DOCX file. [file eneuro-11-ENEURO.0320-24.2024-s006.docx]

| **Relationship** | **Silent Correlation Value (Pearson R or Spearman Rho)** | **P-Value** | **Active R Correlation Value (Pearson R or Spearman Rho)** | **P-Value** |
| --- | --- | --- | --- | --- |
| I_KCA_ v I_A_ | R = -0.7080 | 0.0021 | R = -0.3646 | 0.2206 |
| I_KCA_ v I_KD_ | R = -0.8451 | 0.00001 | R = 0.3978 | 0.1783 |
| I_A_ v I_KD_ | R = 0.7539 | 0.0007 | R = -0.1298 | 0.6724 |

**Table 3-2. LG pairwise correlation values for ionic current relationships.** A relationship was considered to have become less correlated if the silent state R or Rho value was less than -0.6 (P-Value <0.05) or greater than 0.6 (P-Value <0.05) and the active state R or Rho value was less than 0.6 (P-Value >0.05) but greater than -0.6 (P-Value >0.05).
