# Supplementary material for: Distinct Strategies Regulate Correlated Ion Channel mRNAs and Ionic Currents in Continually versus Episodically Active Neurons
Source: eNeuro. 2024 Nov 12;11(11):ENEURO.0320-24.2024. doi: 10.1523/ENEURO.0320-24.2024 (PMC11574698; doi:10.1523/ENEURO.0320-24.2024)
Supplement: Table 4-1 — PD pairwise correlation values for ion channel mRNA relationships. Download Table 4-1, DOCX file. [file eneuro-11-ENEURO.0320-24.2024-s007.docx]

| **Relationship** | **Active Correlation Value (Pearson R)** | | **P-Value** |
| --- | --- | --- | --- |
| *BKKCA* v *SHAL* | R = 0.6294 | 0.0380 | |
| *BKKCA* v *SHAKER* | R = -0.053 | 0.8907 | |
| *BKKCA* v *SHAB* | R = 0.3292 | 0.3259 | |
| *SHAL* v *SHAB* | R = 0.7609 | 0.0106 | |
| *SHAKER* v *SHAB* | R = 0. 5011 | 0.1401 | |

**Table 4-1. PD pairwise correlation values for ion channel mRNA relationships.**
