# Supplementary material for: Distinct Strategies Regulate Correlated Ion Channel mRNAs and Ionic Currents in Continually versus Episodically Active Neurons
Source: eNeuro. 2024 Nov 12;11(11):ENEURO.0320-24.2024. doi: 10.1523/ENEURO.0320-24.2024 (PMC11574698; doi:10.1523/ENEURO.0320-24.2024)
Supplement: Table 4-2 — PD pairwise correlation values for ionic current relationships. Download Table 4-2, DOCX file. [file eneuro-11-ENEURO.0320-24.2024-s008.docx]

| **Relationship** | **Active Correlation Value (Pearson R)** | **P-Value** |
| --- | --- | --- |
| I_KCA_ v I_A_ | R = 0.7387 | 0.0579 |
| I_KCA_ v I_KD_ | R = 0.7273 | 0.0264 |
| I_A_ v I_KD_ | R = 0.6115 | 0.0105 |

**Table 4-2. PD pairwise correlation values for ionic current relationships.**
