# Supplementary material for: Distinct Strategies Regulate Correlated Ion Channel mRNAs and Ionic Currents in Continually versus Episodically Active Neurons
Source: eNeuro. 2024 Nov 12;11(11):ENEURO.0320-24.2024. doi: 10.1523/ENEURO.0320-24.2024 (PMC11574698; doi:10.1523/ENEURO.0320-24.2024)
Supplement: Table 5-1 — Pairwise correlation values for ionic currents vs mRNA relationships. Download Table 5-1, DOCX file. [file eneuro-11-ENEURO.0320-24.2024-s009.docx]

| **Cell type** | **Relationship** | **Correlation Value (Pearson R or Spearman Rho)** | **P-Value** |
| --- | --- | --- | --- |
| PD | I_KCa_ v *BKKCA* | R = -0.1580 | 0.6847 |
| PD | I_A_ v *SHAL* | R = 0.8179 | 0.0038 |
| PD | I_A_ v *SHAKER* | R = -0.7658 | 0.0447 |
| PD | I_Kd_ v *SHAB* | R = -0.3750 | 0.2856 |
| LG silent | I_KCa_ v *BKKCA* | R = 0.4190 | 0.1359 |
| LG silent | I_A_ v *SHAL* | R = -0.2578 | 0.3735 |
| LG silent | I_A_ v *SHAKER* | R = 0.1567 | 0.5928 |
| LG silent | I_Kd_ v *SHAB* | Rho = 0.0991 | 0.7359 |
| LG Active | I_KCa_ v *BKKCA* | Rho = -0.1122 | 0.7152 |
| LG Active | I_A_ v *SHAL* | R = -0.4956 | 0.0850 |
| LG Active | I_A_ v *SHAKER* | R = -0.0754 | 0.8065 |
| LG Active | IKd v *SHAB* | R = -.2107 | 0.4896 |

**Table 5-1. Pairwise correlation values for ionic currents vs mRNA relationships.**
