# Supplementary material for: Distinct Strategies Regulate Correlated Ion Channel mRNAs and Ionic Currents in Continually versus Episodically Active Neurons
Source: eNeuro. 2024 Nov 12;11(11):ENEURO.0320-24.2024. doi: 10.1523/ENEURO.0320-24.2024 (PMC11574698; doi:10.1523/ENEURO.0320-24.2024)
Supplement: Table 6-1 — LG coefficient of variation and Levene's test. COV was calculated for every ionic current measured, and Levene's test p-values were computed to ascertain if variation difference between conditions were significantly different. Download Table 6-1, DOCX file. [file eneuro-11-ENEURO.0320-24.2024-s010.docx]

| **Currents** | | | |
| --- | --- | --- | --- |
|  | **Silent** | **Active** | **Levene’s test (P-value)** |
| I_A_ | 36.9374 | 10.0653 | 0.0063 |
| I_KCa_ | 40.1499 | 16.3385 | 0.0201 |
| I_Kd_ | 50.1866 | 25.9638 | 0.0433 |
| **mRNAs** | | | |
| SHAKER | 28.2117 | 57.9312 | 0.2112 |
| SHAL | 66.3708 | 42.2234 | 0.3411 |
| BKKCa | 46.2522 | 35.7567 | 0.0991 |
| SHAB | 41.1852 | 38.1032 | 0.1144 |

**Table 6-1. LG coefficient of variation and Levene’s test.** COV was calculated for every ionic current and mRNAs measured, and Levene’s test p-values were computed to ascertain if variation difference between conditions were significantly different.
